# Evaluation of fluorine‐19 magnetic resonance imaging of the lungs using octafluorocyclobutane in a rat model

**DOI:** 10.1002/mrm.28473

**Published:** 2020-08-12

**Authors:** Yurii Shepelytskyi, Tao Li, Vira Grynko, Camryn Newman, Francis T. Hane, Mitchell S. Albert

**Affiliations:** ^1^ Chemistry and Materials Science Program Lakehead University Thunder Bay Ontario Canada; ^2^ Thunder Bay Regional Health Research Institute Thunder Bay Ontario Canada; ^3^ Chemistry Department Lakehead University Thunder Bay Ontario Canada; ^4^ Biology Department Lakehead University Thunder Bay Ontario Canada; ^5^ Northern Ontario School of Medicine Thunder Bay Ontario Canada

**Keywords:** fluorine‐19, lung magnetic resonance imaging, octafluorocyclobutane, perfluoropropane

## Abstract

**Purpose:**

To test octafluorocyclobutane (OFCB) as an inhalation contrast agent for fluorine‐19 MRI of the lung, and to compare the image quality of OFCB scans with perfluoropropane (PFP) scans

**Theory and Methods:**

After normalizing for the number of signal averages, a theoretical comparison between the OFCB signal‐to‐noise ratio (SNR) and PFP SNR predicted the average SNR advantage of 90% using OFCB during gradient echo imaging. The OFCB relaxometry was conducted using single‐voxel spectroscopy and spin‐echo imaging. A comparison of OFCB and PFP SNRs was performed in vitro and in vivo. Five healthy Sprague‐Dawley rats were imaged during single breath‐hold and continuous breathing using a Philips Achieva 3.0T MRI scanner (Philips, Andover, MA). The scan time was constant for both gases. Statistical comparison between PFP and OFCB scans was conducted using a paired *t* test and by calculating the Bayes factor.

**Results:**

Spin‐lattice (T_1_) and effective spin‐spin ($T_{2}^{*}$) relaxation time constants of the pure OFCB gas were determined as 28.5 ± 1.2 ms and 10.5 ± 1.8 ms, respectively. Mixing with 21% of oxygen decreased T_1_ by 30% and $T_{2}^{*}$ by 20%. The OFCB in vivo images showed 73% higher normalized SNR on average compared with images acquired using PFP. The statistical significance was shown by both paired *t* test and calculated Bayes factors. The experimental results agree with theoretical calculations within the error of the relaxation parameter measurements.

**Conclusion:**

The quality of the lung images acquired using OFCB was significantly better compared with PFP scans. The OFCB images had higher a SNR and were artifact‐free.

## 1 INTRODUCTION

MRI of inhaled inert fluorinated gases demonstrated promising results as a novel lung imaging modality.^1^, ^2^, ^3^ A variety of studies using sulfur hexafluoride (SF_6_),^4^, ^5^, ^6^ perfluoroethane (C_2_F_6_),^7^, ^8^, ^9^ and perfluoropropane (PFP‐C_3_F_8_)^2^, ^5^, ^10^, ^11^, ^12^ demonstrated the feasibility of fluorine‐19 (^19^F) MRI of the lung for diagnostics and the study of many lung disorders. Fluorinated gases can be mixed with oxygen (O_2_) and used for continuous‐breathing imaging, which allows for dynamic scanning and the study of dynamic lung physiology, including the fractional ventilation measurement study.^4^, ^13^ The short T_1_ relaxation times of fluorinated gases allows a high number of signal averages, resulting in a sufficient image signal‐to‐noise ratio (SNR). Other advantages of ^19^F lung MRI are that it has a high natural abundance (~100%) and a large gyromagnetic ratio, which maximizes the ^19^F MRI signal.^9^

Despite the advantages associated with ^19^F MRI, the SNR of acquired images is lower compared with another lung imaging modality: hyperpolarized noble‐gas MRI.^1^, ^3^ This attribute results from the natural Boltzmann distribution of the spins in the Zeeman energy states for fluorinated gases, as opposed to hyperpolarized gases. Multiple studies have researched ways of improving the quality of ventilation images acquired with fluorinated gases.^2^, ^12^, ^14^, ^15^, ^16^ The main factors that affect SNR are the number of equivalent ^19^F atoms and the relaxation time of the fluorinated gas. Therefore, it is feasible to explore other fluorinated gases that can enhance the SNR associated with ^19^F MRI. Octafluorocyclobutane (C_4_F_8_ [OFCB]) belongs to the family of inert fluorinated gases, contains eight chemically equivalent fluorine atoms per molecule (which is a greater number of equivalent ^19^F atoms compared to other fluorinated gases), and has a longer spin‐spin relaxation time. OFCB is a commercially available gas, with a similar cost as PFP (13.8$ per liter). All these factors make OFCB a promising candidate for ^19^F lung MRI. Although OFCB has not been clinically approved for human inhalation, it has no adverse effects based on inhalation.^17^

Previous reports by Wolf et al.^18^ and Friedrich et al.^19^ used OFCB for the visualization of inert gas washout during high‐frequency oscillatory ventilation. Recently, the first spin‐echo images of human lungs using OFCB were acquired at 0.5T.^20^

The goal of this work was to compare OFCB with PFP and to determine the feasibility of using OFCB as a fluorinated gas for ^19^F lung MRI by comparing its SNR to the SNR of PFP scans. In this work, we demonstrate that OFCB has a higher SNR than PFP for both MR spectroscopy and imaging. In addition, we measured all relaxation parameters of the pure gas and the gas premixed with 20% O_2_ and studied the influence of the unequal number of averages on the SNR comparison.

## 2 THEORY

**FIGURE 1:**
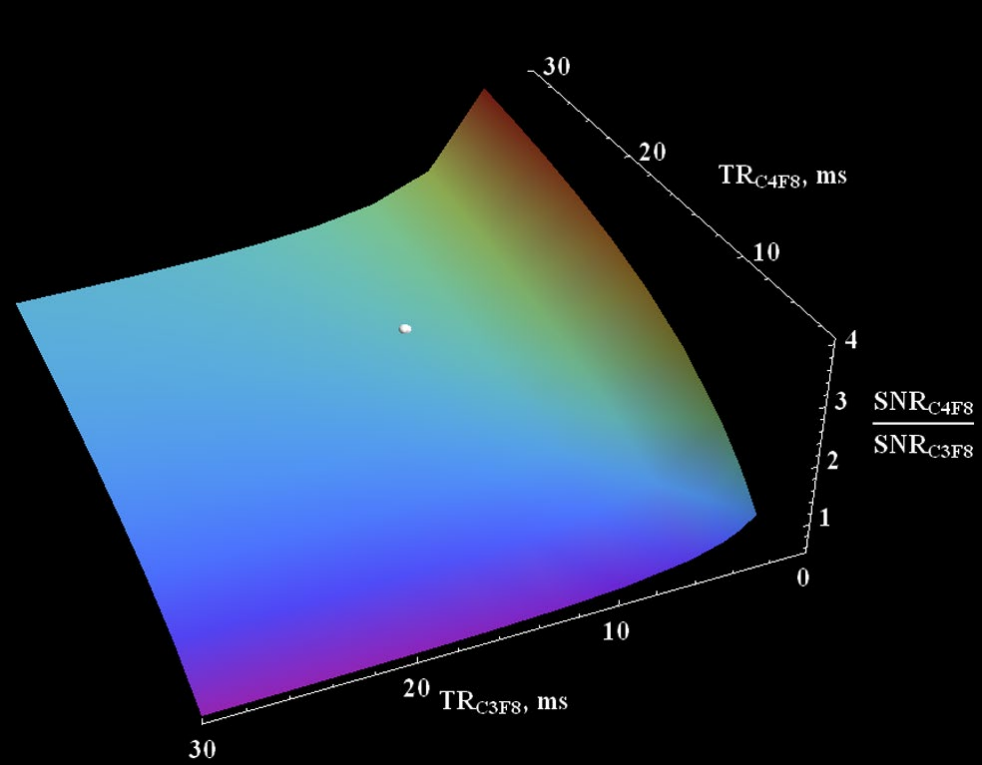
Theoretical dependence of in vivo octafluorocyclobutane–oxygen (OFCB‐O_2_) to perfluoropropane–oxygen (PFP–O_2_) signal‐to‐noise ratio (SNR) as a function of pulse repetition times (TRs). The SNR values were normalized on the number of signal averages (NSA) used for the animal scans (NSA_OFCB_ = 16, NSA_PFP_ = 24). The normalized SNR of PFP gas can excite the normalized SNR of OFCB only if TR_OFCB_ <7 ms. However, this value is impractical for a 70°‐Ernst angle; therefore, it will never be used for the real scans. The white dot represents the experimental results. It can be seen that the experimental result nicely agrees with theoretical calculations

To calculate the theoretical signal for the steady‐state condition, the following equation can be used^2^:

(1) $$S=\;S_{0}\frac{\left(1‐e^{‐\text{TR}/T_{1}}\right)e^{‐\text{TE}/T_{2}^{*}}}{\left(1‐\cos\left(\alpha \right)e^{‐\text{TR}/T_{1}}\right)}\sin\left(\alpha \right),$$

where *α* is the flip angle (FA). Because the T_1_ time of the OFCB–O_2_ mixture is approximately 70% longer compared with PFP–O_2_ (Table 1), to make a proper estimation of their SNR performance, the number of signal averages (NSA) of OFCB–O_2_ scans should be 70% less compared with PFP–O_2_ NSA (to keep scan time the same for both measurements). Using the measured relaxation parameters in vivo (Table 1) and NSA for a single breath‐hold protocol, the ratio of OFCB SNR normalized on NSA to PFP normalized SNR was plotted as a function of pulse repetition time (TR) and echo time (TE) for a 70° FA (Figure 1). Using Equation 1, the theoretical SNR advantage of using OFCB was calculated for three types of scan parameters. For a single breath‐hold experiment, OFCB normalized SNR should be 86% higher compared with PFP. For continuous breathing using a 70° FA, the normalized SNR advantage of OFCB should be equal to 98%, whereas for a full‐recovery regime, the SNR advantage becomes 86%.

**TABLE 1:** Measured T_1_ and $T_{2}^{*}$ relaxation times and gradient image SNR of the studied gases

	T_1_ (ms)	$T_{2}^{*}$, ms	GRE SNR (experimental values)	GRE SNR (normalized for NSA)
OFCB	28.5 ± 1.2	10.5 ± 1.8	45.52	45.52
PFP	18.6 ± 0.4	6.26 ± 0.27	30.26	30.26
OFCB‐O_2_	20.4 ± 0.21	8.6 ± 0.5	14.52	14.52
PFP‐O_2_	14.98 ± 0.61	5.4 ± 0.3	9.42	9.42
OFCB‐O_2_ (in vivo)	17.77 ± 1.5	3.4 ± 0.4	9.72 ± 2.1 (breath‐hold)	0.61 ± 0.13 (breath hold)
			14.48 ± 4.51 (continuous breathing, 70°)	0.1 ± 0.03 (continuous breathing, 70°)
			10.23 ± 0.70 (continuous breathing, 90°)	0.39 ± 0.03 (continuous breathing, 90°)
PFP‐O_2_ (in vivo)	12.8 ± 1.1	2.2 ± 0.3	7.66 ± 2.0 (breath‐hold)	0.32 ± 0.08 (breath hold)
			12.68 ± 4.09 (continuous breathing, 70°)	0.06 ± 0.02 (continuous breathing, 70°)
			8.81 ± 0.46 (continuous breathing, 90°)	0.21 ± 0.01 (continuous breathing, 90°)

Abbreviations: GRE, gradient echo; NSA, number of signal averages; O_2_, oxygen; OFCB, octafluorocyclobutane; PFP, perfluoropropane; SNR, signal‐to‐noise ratio.

## 3 METHODS

### 3.1 General information

This study was divided into two parts: (1) A phantom study to measure the relaxation parameters of pure gases and O_2_ mixtures. A SNR comparison of OFCB and PFP gradient echo (GRE) images was also conducted. (2) A SNR comparison in vivo by acquiring ventilation images of healthy rat lungs. For this study, a clinical Philips Achieva 3T MRI scanner (Philips, Andover, MA) was equipped with a custom‐built quadrature birdcage coil tuned to the Larmor frequency of fluorine (120.15 MHz). Four phantoms, consisting of a syringe containing 8 mL of one of the gases, OFCB (99.9999%; Advanced Specialty Gases, Reno, NV), pure PFP (>99.99%; Air Liquide, Paris, France), OFCB breathing mixture (79% OFCB mixed with 21% O_2_), and the medical‐grade PFP (79% PFP mixed with 21% O_2_) were used.

### 3.2 Phantom study

MR spectra of the gas phantoms were acquired using the following parameters: TR/TE = 750 ms/0.14 ms, bandwidth (BW) = 32 kHz, sampling number = 2048, and FA = 90°. The spectral peaks were fitted to the Lorentzian peak shape and $T_{2}^{*}$ was extracted from full‐width half‐maximum (FWHM) of the fitted peak using equation $T_{2}^{*}$ = 1/πFWHM.

To measure the spin‐lattice (T_1_) relaxation time constant, a series of inversion recovery (IR) spectra was acquired. Pure gases were studied using the following inversion times (TIs): TI_min_ = 4 ms, TI_max_ = 91 ms, and ΔTI = 3 ms. The O_2_ mixtures were studied using TI_min_ = 4 ms, TI_max_ = 28 ms, and ΔTI = 1 ms. Other spectroscopy parameters were the same as outlined above.

Following the spectroscopy study, the direct comparison of the two axial ^19^F GRE images of OFCB phantoms and PFP phantoms were acquired. The following GRE imaging parameters were used for the imaging of pure gases: field of view (FOV) = 100 × 100 mm^2^, 64 × 64 matrix, TR/TE = 200 ms/1 ms, and Cartesian sampling. To image the breathing mixture phantoms, the following repetition times were used: TR_PFP‐O2_ = 63 ms, TR_OFCB‐O2_ = 100 ms, and FA = 90°. All other parameters were kept the same for imaging the pure gases. The SNR was calculated as the peak intensity to the standard deviation (SD) of the noise region ratio.

### 3.3 Animal study

#### 3.3.1 Animal preparation

All animal studies were conducted in accordance with the guidelines of the Canadian Council on Animal Care and approved by the Lakehead University Animal Care Committee (AUP 1463772). Five healthy Sprague‐Dawley rats weighing between 300 and 400 g were imaged in this study. The animals were prepared for surgery as described in Chahal et al.^21^ Briefly, rats were anesthetized with isoflurane and propofol. A midline incision allowed an endotracheal catheter to be placed. The catheter was connected to a custom‐built rodent ventilator.

The rat was given a OFCB–O_2_ breathing mixture (79% of OFCB mixed with 21% O_2_) at 60 breaths per minute with a 4‐mL tidal volume. The rat was placed inside the custom‐built quadrature ^19^F coil. After the OFCB data acquisition, the ventilator was switched to pure O_2_ to remove any OFCB left inside the lungs. Following 5 minutes of O_2_ ventilation, the ventilator was switched to a PFP–O_2_ breathing mixture. Following the PFP data acquisition, the animals were euthanized by barbiturate overdose.

#### 3.3.2 In vivo imaging

Two different breathing protocols were performed in this study: a single breath‐hold for 11 seconds, and continuous breathing for 3 minutes and 5 seconds. All lung images were acquired using a GRE pulse sequence with a Cartesian readout. All animals were scanned during a single breath‐hold; however, only three rats were scanned using the continuous breathing protocol. During continuous breathing, the two sets of scans were conducted: (1) using the Ernst angle condition that is most commonly used in preclinical studies, and (2) using the condition of full recovery of longitudinal magnetization for a more accurate comparison between two gases at laboratory conditions because this regime is almost insensitive to T_1_ variation of the inhaled gas mixture.

T_1_ and $T_{2}^{*}$ relaxation times have been measured in vivo using the same approach from the phantom study.

The ^19^F lung projection images during single breath‐hold were acquired using the following parameters: FOV = 100 × 100 mm^2^, 32 × 32 acquisition matrix, TE = 0.63 ms, FA = 70°, and BW = 436 Hz/pixel. To keep the scan time equal to the breath‐hold duration, the NSAs were equal to 16 and 24 for OFCB and PFP breathing mixtures, respectively.

The ^19^F lung projections for the continuous breathing protocol were acquired either using full recovery (FA = 90°) or using the 70° Ernst FA. The following GRE pulse sequence parameters were used: FOV = 100 × 100 mm^2^, 64 × 64 acquisition matrix, TE = 0.95 ms, BW = 246 Hz/pixel, and scan time = 185 seconds. The NSAs of 144 and 221 were used for OFCB and PFP, respectively, when the 70° FA was used. During the full‐recovery scans, the NSA for the OFCB scan was equal to 29, whereas the PFP NSA was equal to 41. The following TR values were used in this study: TR_PFP‐O2_/TR_OFCB‐O2_ = 12.5 ms/20 ms (FA = 70°) and TR_PFP‐O2_/TR_OFCB‐O2_ = 63 ms/100 ms (FA = 90°). No respiratory gating was used.

#### 3.3.3 Data processing

The spectroscopy data processing, paired *t* test, and all fitting were calculated using OriginPro 2016 software (OriginLab Corp, Northampton, MA). The ^19^F MR images were reconstructed and analyzed using custom MATLAB scripts in MATLAB R2016b (MathWorks, Inc, Natick, MA). The image SNR was calculated as the mean signal value in a rectangular region of interest in the right lung divided by the SD of noise in a similar region of interest in the background. The calculation of the Bayes factor for the statistical analysis was conducted using the MATLAB Bayes factor package (v.1.0.0 by Bart Krekelberg). The criterion of significance of the results, based on the value of Bayes factor, was used as published by Kass and Raftery.^22^ The theoretical TR versus TE plot was created using Wolfram Mathematica 9.0.1.0 software (Wolfram Research, Inc, Champaign, IL).

## 4 RESULTS

### 4.1 The phantom study

**FIGURE 2:**
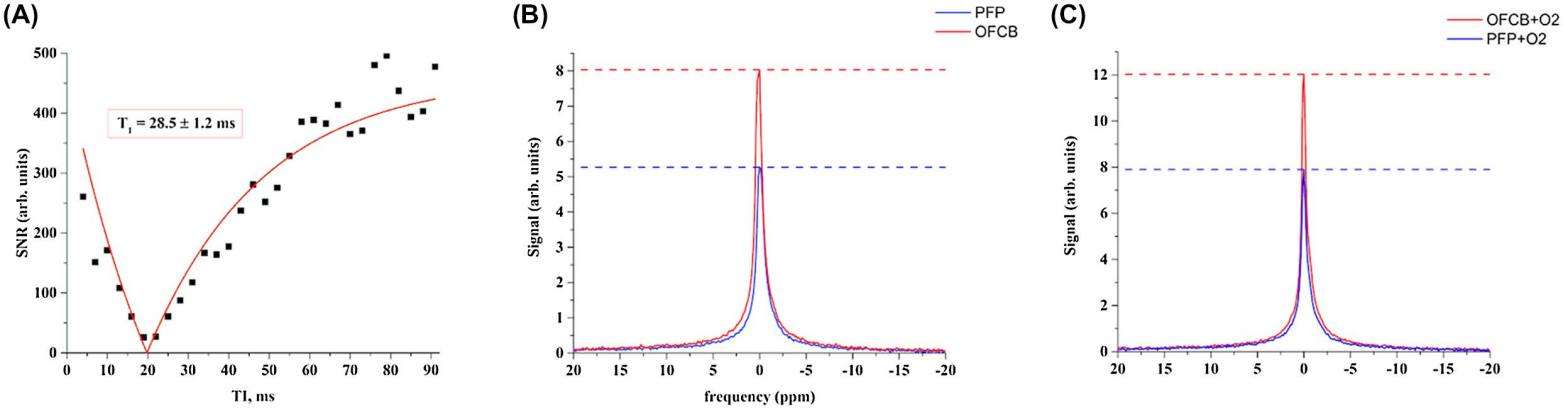
A, A representative inversion recovery curve measured for pure octafluorocyclobutane (OFCB). B, The measured spectra of 8 mL of pure perfluoropropane (PFP; blue) and OFCB (red). C, Spectra were obtained from 8 mL of PFP and OFCB breathing mixtures. The horizontal lines represent the maximum value of the corresponding magnetic resonance spectroscopy peak

The spin‐lattice relaxation time constant (T_1_) of pure OFCB was measured to be 28.5 ± 1.2 ms (Figure 2A). The T_1_ relaxation time of pure PFP was measured and was equal to 18.6 ± 0.4 ms, which is similar to values reported in Chang and Conradi.^23^ The measured $T_{2}^{*}$ relaxation times were equal to 10.5 ± 1.8 ms and 6.26 ± 0.3 ms for OFCB and PFP, respectively.

Following relaxometry of the pure gases, the relaxation properties of the 20% O_2_ mixtures were measured. The T_1_ relaxation time of the OFCB–O_2_ mixture was shortened to 20.4 ± 0.21 ms, and the T_1_ time of the PFP–O_2_ mixture was equal to 14.98 ± 0.61 ms. The $T_{2}^{*}$ relaxation times were equal to 8.6 ± 0.5 ms and 5.4 ± 0.3 ms for OFCB–O_2_ and PFP–O_2_ mixtures, respectively.

Single‐voxel (SV) spectroscopy of OFCB, PFP, and their respective O_2_ mixtures was conducted to see the signal difference on the MR spectra. The acquired spectra of the pure gases are shown in Figure 2A. The single‐voxel spectra of the 20% O_2_ mixtures are presented in Figure 2C. The SNR values were equal to 628.44, 499.91, 400.44, and 362.10 for the OFCB, OFCB–O_2_, PFP, and PFP–O_2_ phantoms, respectively. The SNR value obtained from pure OFCB gas was approximately 1.57 times higher than pure PFP SNR. However, the SNR value of the OFCB–O_2_ spectrum was approximately 38% higher than the PFP–O_2_ SNR.

Because the PFP gas has a shorter $T_{2}^{*}$ relaxation time, the peak appeared broader and shorter. The ratio of PFP integral values to the OFCB integral was equal to 0.74 and 0.75 for pure gases and O_2_ mixtures. This result agrees with the theoretical 6:8 ratio predicted from a molecular structure of the studied gases.

GRE imaging was conducted on phantoms to evaluate the SNR performance of OFCB. The OFCB images were compared with the image of the main peak of PFP. The SNR of the pure PFP phantom image was equal to 30.26, whereas the SNR of the pure OFCB was approximately 50% higher and equal to 45.52. The presence of O_2_ did not cause any decrease for the SNR difference. The SNR of the OFCB–O_2_ mixture (SNR = 14.22) was 51% higher than the SNR of the medical‐grade PFP (SNR = 9.42). The measured relaxation times and SNR values are summarized in Table 1.

### 4.2 The animal study

**FIGURE 3:**
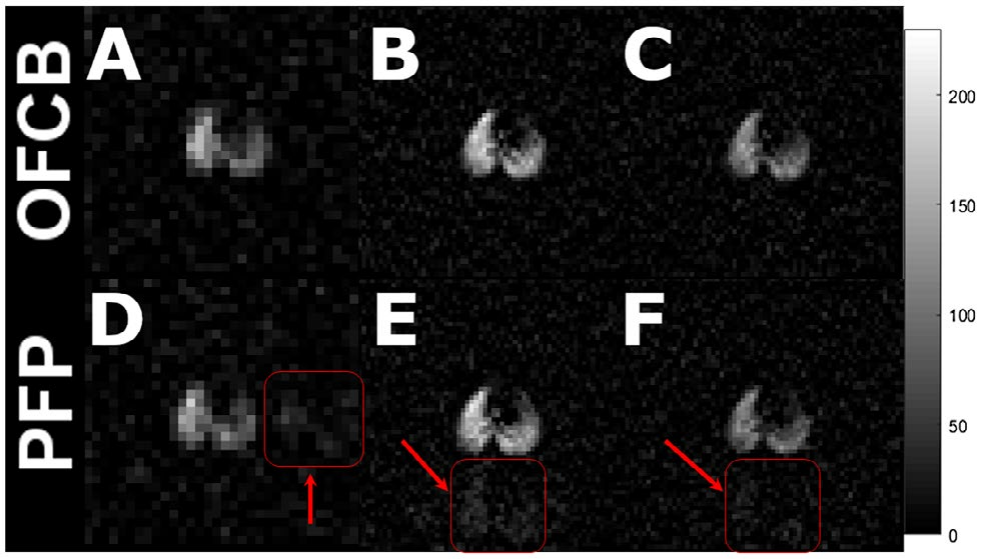
In vivo lung ventilation images of a healthy rat acquired in axial projections. The first column shows scans acquired during a single breath‐hold; the second column corresponds to the scans acquired during continuous breathing and using a 70°‐Ernst angle; the third column contains scans obtained during continuous breathing using a 90° flip angle (FA). The signal‐to‐noise ratio (SNR) of the single breath‐hold octafluorocyclobutane (OFCB) scan was 21% higher compared with the corresponding perfluoropropane (PFP) scan. For continuous breathing, the SNR of the OFCB image acquired using the Ernst angle of 70° was 15% stronger. Finally, during continuous breathing scans in the full recovery regime, the OFCB SNR exceeded PFP SNR by 17%. The red arrows indicate the chemical shift artifact associated with second spectral peak of PFP

Figure 3 shows whole‐lung projections in the axial plane acquired during a single breath‐hold (Figure 3A,D) and continuous breathing (Figure 3B,C,E,F) from the same animal. The first row of images shows the OFCB scans, and the second row shows the PFP scans. Figure 3B and E were acquired using FA = 70°, TR_PFP_ = 13 ms, and TR_OFCB_ = 20 ms. Figure 3C and F were acquired using a full recovery of longitudinal magnetization condition.

The normalized SNR value for the NSA of the OFCB single breath‐hold image was equal to 0.61, which was approximately 85% larger than the normalized SNR image from the PFP breathing mixture (SNR = 0.33). The normalized SNR values of images acquired using a FA = 70° during 185 seconds of continuous breathing were equal to 0.11 and 0.06 for OFCB–O_2_ and PFP–O_2_ mixtures, respectively. The SNR advantage of using OFCB was calculated to be 83%. Finally, the images acquired using a full‐recovery condition during continuous breathing had a normalized SNR of 0.37 and 0.22 for OFCB and PFP, respectively.

The T_1_ values of OFCB–O_2_ and PFP–O_2_ mixtures in the animal lungs were equal to 17.77 ± 1.5 ms and 12.8 ± 1.1 ms. $T_{2}^{*}$ values were equal to 3.4 ms and 2.2 ms for OFCB–O_2_ and PFP–O_2_ breathing mixtures, respectively.

**FIGURE 4:**
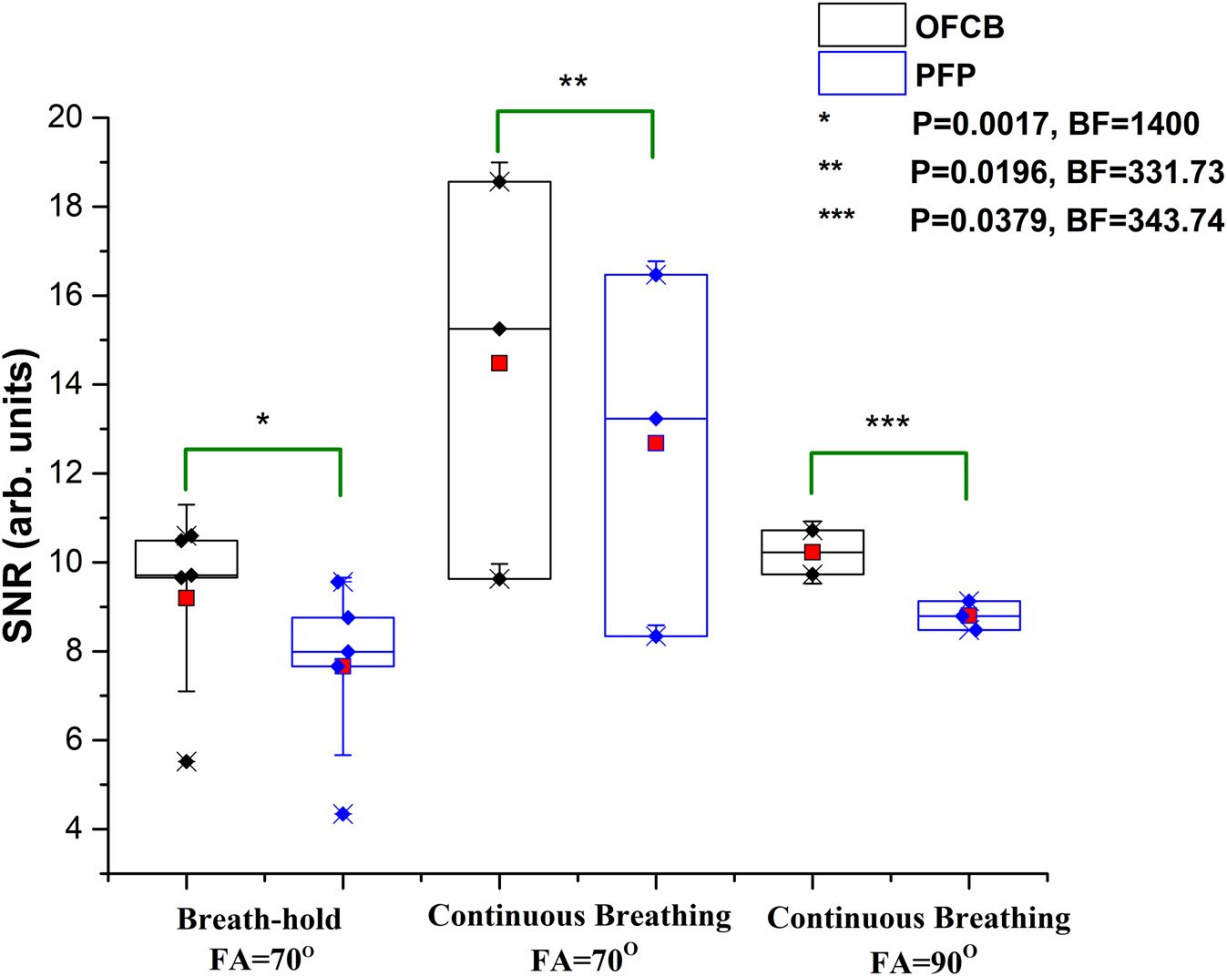
Non‐normalized signal‐to‐noise ratio (SNR) box charts of scans conducted using the single breath‐hold protocol, and continuous breathing protocol. The black boxes correspond to octafluorocyclobutane (OFCB) scans, and the blue boxes correspond to perfluoropropane (PFP) scans. The red squares illustrate the mean SNR of the group. Whiskers illustrate the standard deviation from the mean SNR. The SNR values of the OFCB scans were statistically significantly higher compared with the PFP scans. The corresponding *P* values and Bayes factors are shown in the figure legend. The wider scatter of SNR values obtained using the Ernst angle condition during continuous breathing can be explained by the absence of respiratory gating. The Ernst angle condition strongly depends on the T_1_ relaxation time of the gas in the lungs, and the absence of gating caused a T_1_ variation because of the fluorinated gas‐concentration differences during imaging

The nonnormalized SNR values of images acquired using a single breath‐hold protocol and the SNR values of the scans acquired during the continuous‐breathing protocol using a FA = 70° and a FA = 90° are shown in Figure 4. The SNR of the one axial ventilation image acquired during continuous breathing using a 90° FA was not calculated because a ghosting artifact was observed on the image. Therefore, this scan was excluded from further statistical analysis. The mean normalized and non‐normalized SNR values are provided in Table 1.

### 4.3 Statistical analysis

A paired *t* test was used to evaluate the SNR difference between the OFCB and PFP scans for each image acquisition protocol. The mean values of the nonnormalized SNR values for single breath‐hold images were equal to 9.12 ± 2.10 and 7.66 ± 2.00 for OFCB and PFP breathing mixtures, respectively. The OFCB produced significantly a higher SNR (*P* = .0017), which was supported by a Bayes coefficient of 1.4 × 10^3^.

The mean noncorrected SNR of OFCB images obtained during continuous breathing were equal to 14.48 ± 4.51 and 10.23 ± 0.7 using a FA = 70° and a FA = 90°, respectively. The average SNR values of PFP images were equal to 12.68 ± 4.09 (FA = 70°) and 8.81 ± 0.46 (FA = 90°). OFCB SNR values were significantly higher compared with PFP values (*P* = .0196 [FA = 70°]; *P* = .038 [FA = 90°]). This significance was supported by values of the Bayes coefficient: 331.73 (FA = 70°) and 343.74 (FA = 90°). Both normalized and non‐normalized SNR values are provided in Table 1.

## 5 DISCUSSION

Inert fluorinated gases can be used as gas contrast agents for MRI of the lungs. Currently, PFP is the most common gas agent used in preclinical studies.^1^, ^3^ The results presented above demonstrate the benefits of using OFCB gas as an MRI contrast agent. OFCB is inert, which makes it safe for inhalation. The main advantage of OFCB over PFP is the presence of eight chemically equivalent nuclei in the molecule. Furthermore, it has a longer effective transverse relaxation time constant than the PFP $T_{2}^{*}$ value. These two properties cause OFCB SNR to be 1.57 times higher than the PFP SNR. The results comparing GRE SNR of PFP and OFCB phantoms showed slightly less SNR differences than spectroscopy. However, the SNR of OFCB images was significantly higher than images of PFP phantoms.

The measured spin‐lattice relaxation parameter of pure OFCB was similar to that reported by Friedirich et al.^19^ Interestingly, the effective spin‐spin relaxation time constant was approximately half of what was previously published. This shortening of $T_{2}^{*}$ could be explained by the effect of a two times stronger external magnetic field compared to what was previously used in the literature.^19^ The obtained PFPs relaxation parameters values were close to those published by Chang and Couch.^23^, ^24^

The short T_1_ relaxation time is an advantage of fluorinated gases, which allows high NSA acquisition during a single breath‐hold. OFCB has the longer T_1_ relaxation time compared with other widely used fluorinated gases. Theoretical calculations of SNR showed that OFCB still produces higher SNR even with a smaller amount of averages. Because OFCB allows the acquisition of higher SNRs using lower NSA, the specific absorption rate of the imaging sequence with OFCB will be lower than for imaging with any other inert fluorinated gas.

OFCB has another practical advantage compared with PFP: The ^19^F spectrum of OFCB contains only one single peak, whereas the PFP spectrum has two peaks. As a result, the signal of the second PFP peak should be suppressed to avoid creation of a second lung image, which could overlap with the image of the main peak. OFCB does not have this drawback, which makes it more convenient for practical applications.

The results of the animal experiments agree with the theoretical calculations (Figure 1). The normalized for NSA OFCB SNR advantage was equal to 90% for a single breath‐hold (white point on Figure 1). The theoretically predicted value of the normalized SNR advantage was 86%. There is a slight deviation from the theory for a continuous breathing protocol. The normalized SNR advantage of OFCB was calculated to be equal to 98% and 86% for the 70°‐Ernst angle and full longitudinal magnetization conditions, respectively. The observed normalized OFCB SNR boosts were equal to 76% for the Ernst‐angle condition and to 86% for the full longitudinal magnetization recovery condition, which is lower than the theoretically predicted value. This can be explained by a slight mismatch between the OFCB T_1_ in vivo and the TR used during the scans and the absence of respiratory gating. The larger scatter of SNR values for the Ernst‐angle condition (Figure 4) is caused by the absence of respiratory gating during the scan. Because the 70°‐Ernst‐angle condition depends strongly on the T_1_ of the gas in the lungs, the absence of respiratory gating can potentially cause a variation of T_1_ based on the different concentrations of the fluorinated gases in the lungs. All of the three predicted advantages of OFCB (higher SNR, absence of chemical shift artifacts, low specific absorption rate) were observed. The SNR of OFCB images was significantly higher even with an approximately 65% smaller number of signal averages.

In this study, we showed that OFCB is a suitable candidate for ^19^F MRI of the lungs. The image quality of OFCB scans was significantly higher compared with commonly used PFP. In addition, OFCB scans are safer in terms of tissue‐heating because of a lower specific absorption‐rate value compared with PFP. The roughly estimated OFCB‐scan specific absorption rate is approximately 60% lower compared with PFP scans because of the smaller number of signal averages.
